# Loss of the Antimicrobial Peptide Metchnikowin Protects Against Traumatic Brain Injury Outcomes in *Drosophila melanogaster*

**DOI:** 10.1534/g3.120.401377

**Published:** 2020-07-06

**Authors:** Laura C. Swanson, Stacey A. Rimkus, Barry Ganetzky, David A. Wassarman

**Affiliations:** * Department of Medical Genetics, School of Medicine and Public Health, University of Wisconsin-Madison, Madison, WI 53706; † Cellular and Molecular Biology Graduate Program, University of Wisconsin-Madison, Madison, WI 53706; ‡ Medical Scientist Training Program, School of Medicine and Public Health, University of Wisconsin-Madison, Madison, WI 53706; § Department of Genetics, College of Agricultural and Life Sciences, University of Wisconsin-Madison, Madison, WI 53706

**Keywords:** antimicrobial peptide, *Drosophila melanogaster*, innate immune response, Metchnikowin, traumatic brain injury

## Abstract

Neuroinflammation is a major pathophysiological feature of traumatic brain injury (TBI). Early and persistent activation of innate immune response signaling pathways by primary injuries is associated with secondary cellular injuries that cause TBI outcomes to change over time. We used a *Drosophila melanogaster* model to investigate the role of antimicrobial peptides (AMPs) in acute and chronic outcomes of closed-head TBI. AMPs are effectors of pathogen and stress defense mechanisms mediated by the evolutionarily conserved Toll and Immune-deficiency (Imd) innate immune response pathways that activate Nuclear Factor kappa B (NF-κB) transcription factors. Here, we analyzed the effect of null mutations in 10 of the 14 known *Drosophila* AMP genes on TBI outcomes. We found that mutation of *Metchnikowin* (*Mtk*) was unique in protecting flies from mortality within the 24 h following TBI under two diet conditions that produce different levels of mortality. In addition, *Mtk* mutants had reduced behavioral deficits at 24 h following TBI and increased lifespan either in the absence or presence of TBI. Using a transcriptional reporter of gene expression, we found that TBI increased *Mtk* expression in the brain. Quantitative analysis of mRNA in whole flies revealed that expression of other AMPs in the Toll and Imd pathways as well as NF-κB transcription factors were not altered in *Mtk* mutants. Overall, these results demonstrate that *Mtk* plays an infection-independent role in the fly nervous system, and TBI-induced expression of *Mtk* in the brain activates acute and chronic secondary injury pathways that are also activated during normal aging.

Traumatic brain injury (TBI) is a leading cause of death and disability worldwide, affecting over 50 million individuals each year (Maas *et al.* 2017). Survivors of TBI often suffer a range of physical and neurological impairments, including cognitive and psychosocial disorders, post-traumatic epilepsy, and increased incidence of dementia and neurodegeneration (Zaloshnja *et al.* 2008; Bazarian *et al.* 2009; Chauhan 2014; Diamond *et al.* 2015; Juengst *et al.* 2017; Ng and Lee 2019). TBI pathophysiology results from both primary and secondary injuries. Primary injuries are a consequence of closed-head or penetrating mechanical forces on the brain that cause direct physical damage to brain cells (Ng and Lee 2019). Secondary injuries begin immediately following primary injuries and are the consequence of cellular and molecular responses to primary injures that create damage at and beyond the site of the primary injury (Ray *et al.* 2002; Kumar Sahel *et al.* 2019; Ng and Lee 2019). Understanding the mechanisms by which secondary injury-inducing pathways influence TBI outcomes should provide opportunities for therapeutic intervention (Somayaji *et al.* 2018).

Neuroinflammation plays a major role in initiation and progression of secondary injuries following TBI (Kumar and Loane 2012; Lozano *et al.* 2015; Kumar Sahel *et al.* 2019). Both clinical and animal studies of TBI have revealed rapid recruitment of pro- and anti-inflammatory cytokine- and chemokine-secreting microglia to sites of damaged tissue, which can persist for days to years following an injury (Acosta *et al.* 2013a, 2013b; Hernandez-Ontiveros *et al.* 2013; Lozano *et al.* 2015; Long *et al.* 2020). Although high levels of inflammation correlate with detrimental TBI outcomes, inflammation also can be neuroprotective, suggesting that approaches aimed at regulating TBI-induced inflammation will need to be targeted in precise ways (Schmidt *et al.* 2005; Russo and McGavern 2016). To date, no therapies have had a substantial impact when moved to clinical trials (Tortella 2016; Chen *et al.* 2017; Xu *et al.* 2017; DeWitt *et al.* 2018). A possible contributing factor to the lack of success is that it is difficult to replicate in mammalian TBI models the inherent heterogeneity in primary injuries as well as genetic and environmental factors that affect secondary injuries in humans (Ganetzky and Wassarman 2016; Alves *et al.* 2019).

To identify genetic and environmental factors that affect TBI outcomes, we developed a high-throughput method of inflicting closed-head TBI in *Drosophila melanogaster* (Katzenberger *et al.* 2013, 2015c). We found that TBI in flies shares key characteristics with TBI in mammals, including temporary incapacitation, short-term ataxia, progressive neurodegeneration, intestinal barrier dysfunction, hyperglycemia, and shortened lifespan (Katzenberger *et al.* 2013, 2015a). Furthermore, consistent with findings of an activated Nuclear Factor kappa B (NF-κB) response in mammals, transcriptional gene targets of the homologous NF-κB-mediated Toll and Immune-deficiency (Imd) signaling pathways are rapidly and persistently activated following TBI in multiple fly models (Katzenberger *et al.* 2013, 2015a, 2015b 2016; Barekat *et al.* 2016; Ratliff *et al.* 2016; Sen *et al.* 2017; Shah *et al.* 2019). For example, expression of major Toll and Imd gene targets, antimicrobial peptides (AMPs), substantially increases within 30 min after TBI and remains elevated for at least 24 h (Katzenberger *et al.* 2016). AMPs are structurally diverse peptides that defend eukaryotic hosts from bacterial and fungal pathogens (Zhang and Gallo 2016). In addition, recent studies have shown that AMPs have functions in the absence of infection (Hanson and Lemaitre 2020). In Alzheimer’s disease (AD) brains, AMPs are highly expressed in glial cells, and a key protein in AD pathology, amyloid-β peptide, has antimicrobial activity (Soscia *et al.* 2010; Kagan *et al.* 2012; Kumar *et al.* 2016; Spitzer *et al.* 2016; Tsuda and Lim 2018). In *C. elegans*, a particular AMP triggers aging-dependent dendrite degeneration by serving as a ligand for a neuronal G protein-coupled receptor (E *et al.* 2018). In flies, misexpression of AMP genes in the nervous system causes neurodegeneration, behavioral defects, and a shortened lifespan, while suppression of the Imd pathway in glial cells increases lifespan and reduces behavioral deficits (Cao *et al.* 2013; Kounatidis *et al.* 2017). Furthermore, endogenous bacteria do not appear to drive acute TBI outcomes in flies, as the risk of early mortality and intestinal barrier dysfunction is not different between bacteria-containing and bacteria-free flies (Katzenberger *et al.* 2015a). We are interested in determining whether the increase in AMP gene expression that occurs following a primary injury has a causal effect on TBI outcomes.

Recently, a set of *Drosophila* AMP gene knockout lines was developed to explore the effect of loss of expression of individual or groups of AMP genes on the response to bacterial and fungal pathogens (Hanson *et al.* 2019). We used these lines to identify AMP genes that are necessary for acute and/or chronic outcomes of TBI. Our study revealed that a null mutation of the gene encoding the Toll- and Imd-regulated antifungal peptide Metchnikowin (Mtk) suppressed acute behavioral deficits and mortality as well as lifespan reduction caused by TBI (Levashina *et al.* 1998). Expression of *Mtk* increased in the brain following TBI. Furthermore *Mtk* mutant flies were able to mount an otherwise normal innate immune response following TBI, as indicated by expression of other genes in the Toll and Imd pathways. These data suggest that Mtk functions through a pathogen-independent mechanism in the brain to promote neuropathologies following TBI.

## Materials And Methods

### Fly lines and culturing

**Figure 1:**
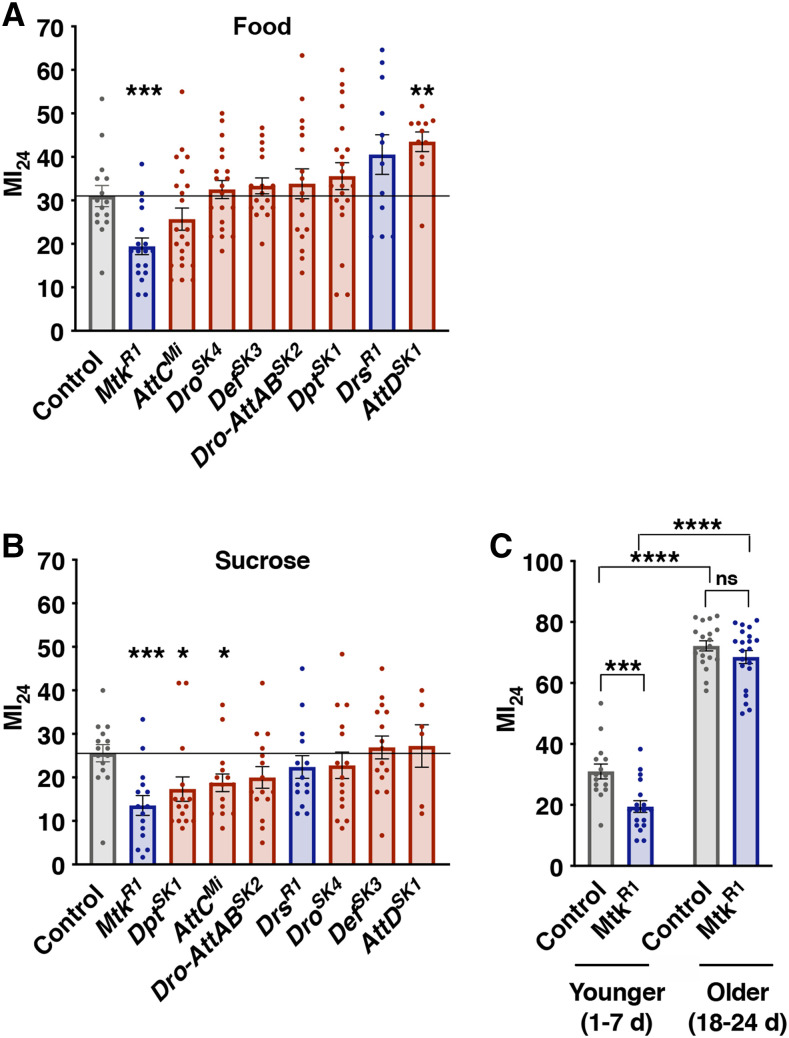
Acute mortality following TBI in flies is reduced in *Mtk*^*R1*^ mutants. The MI_24_ of 1-7 day old AMP mutant flies fed (A) cornmeal-molasses food (food) or (B) 0.4 M sucrose (sucrose) following TBI. Gray bars represent control flies, and blue and red bars represent flies carrying mutations in AMP genes that are primarily activated by the Toll pathway or Imd pathway, respectively. (C) The MI_24_ of control and *Mtk*^*R1*^ flies injured at younger (1-7 day old, 1-7d) or older (18-24 day old, 18-24d) ages. Each bar represents the average of ≥3 independent trials, and each dot represents the outcome for an individual vial of flies. Error bars represent the SEM. Significance for panels A and B was determined by a Student’s unpaired *t*-test, and significance for panel C was determined by a two-way ANOVA with a Bonferroni post-hoc test. *P < 0.05, **P < 0.01, ***P < 0.001, ****P < 0.0001, ns (not significant).

Flies were maintained on cornmeal-molasses food at 25°. The food contained 30 g Difco granulated agar (Becton-Dickinson, Sparks, MD), 44 g YSC-1 yeast (Sigma, St. Louis, MO), 328 g cornmeal (Lab Scientific, Highlands, NJ), 400 ml unsulfured Grandma’s molasses (Lab Scientific), 3.6 L water, 40 ml propionic acid (Sigma), and tegosept (8 g Methyl 4-hydroxybenzoate in 75 ml of 95% ethanol) (Sigma). In Figure 1B, flies were fed sucrose by placing 200 μl of 0.4 M sucrose (Sigma) on a filter paper disk at the bottom of a vial.

AMP mutant lines used in this study were: *Attacin C* (*AttC*^*Mi*^), *Attacin D* (*AttD*^*SK1*^), *Defensin* (*Def*^*SK3*^), *Drosocin* (*Dro*^*SK4*^), *Drosomycin* (*Drs*^*R1*^), and *Metchnikowin* (*Mtk*^*R1*^); two small deletions removing both *Diptericins **DptA* and *DptB* (*Dpt^SK1^*); and a deletion containing *Dro*, *AttA*, and *AttB* (*Dro**-AttAB^SK2^*). We also analyzed a double AMP mutant (*Mtk*^*R1*^;*Drs*^*R1*^). AMP gene mutant lines were generated by CRISPR/Cas9 gene editing (*AttD*^*SK1*^, *Def*^*SK3*^, *Dro*^*SK4*^, and *Dro**-AttAB^SK2^*), *P*-element-mediated homologous recombination (*Mtk*^*R1*^ and *Drs*^*R1*^), or a Minos transposable element (*AttC*^*Mi*^), as described in Hanson *et al.* (2019). Non-CRISPR/Cas9 generated lines were backcrossed to the control line (DrosDel iso *w^1118^*) for at least seven generations to generate a consistent genetic background. All AMP gene mutants and control DrosDel iso *w^1118^* lines were provided by Bruno Lemaitre (École Polytechnique Fédérale de Lausanne, Lausanne, Switzerland). The AMP transcriptional reporter lines Metchnikowin-GFP (Mtk-GFP), Attacin C-GFP (AttC-GFP), and Defensin-GFP (Def-GFP) are described in Tzou *et al.* 2000 and were provided by Ylva Engstrom (Stockholm University, Stockholm, Sweden).

### TBI assay

Flies were injured using the HIT device as described in Katzenberger *et al.* 2013, 2015c. For all experiments using the AMP mutant lines, flies were injured by four strikes at 10 min intervals with the spring deflected to 90°. All vials contained 60 flies (30 males and 30 females) of the indicated age. The average mortality at 24 h for uninjured flies did not exceed 1.8%.

### Climbing and longevity assays

Climbing assays were performed with ten 2-8 day old flies (5 males and 5 females) per vial and was measured at 24 h following TBI. Climbing was assessed by quantifying the percent of flies that failed to climb at least 5 cm in 10 sec after being gently tapped to the bottom of an empty vial. For each vial, the percent failed climbing was averaged between three trials conducted over 30 min.

Longevity was performed with 2-8 day old flies that survived 24 h following TBI. At least five vials containing 20 flies (10 males and 10 females) were examined per condition, each with three independent biological replicates (n ≥ 150 per sex). Vials were maintained at 25°. Every three days, flies were transferred to fresh food vials and the number of living flies was recorded. Percent survival was averaged among vials for each condition.

### Brain dissection and immunostaining

5-6 day old Mtk-GFP, AttC-GFP, and Def-GFP homozygous flies were subject to four strikes from the HIT device at 5 min intervals with the spring deflected to 90°. 5 min after TBI, flies were transferred to new vials with a 1-inch Whatman filter paper disk placed in the bottom that contained 200 µl of water. Flies recovered at 25° for 1 h (Def-GFP), 3 h (AttC-GFP), or 4 h (Mtk-GFP) before the brains were dissected. Recovery times were selected based on the peak level of post-TBI expression of the AMP gene, as determined in Katzenberger *et al.* 2016. Approximately 16 brains were dissected for each treatment. Dissected brains were incubated in fresh ice cold 4% formaldehyde in 1X PBS and transferred to a 1.5 ml Eppendorf tube containing 1 ml 4% formaldehyde on ice for 30 min. Brains were washed three times for 20 min in 1.5 ml 1X PBS containing 0.3% Triton-X (PBST) and blocked 20 h at 4° in 150 µl PBST containing 5% normal goat serum. The block solution was removed, and primary antibody solution was added and incubated for 20 h at 4°. Brains were washed three times for 20 min in 1.5 ml PBST at room temperature. Secondary antibody solution was added and incubated for 20 h at 4°. Brains were washed three times for 20 min in 1.5 ml PBST at room temperature, with the second wash containing 1 µg/ml DAPI (Santa Cruz Biotechnology). All washes and incubations were performed in a light-proof box on a shaker. Brains were mounted in Vectashield (Vector Laboratories), and the central brain was imaged at 100X magnification on a Nikon A1R-SI+ confocal microscope (Optical Imaging Core, University of Wisconsin, Madison, WI). All images were taken in a single plane. Primary antibodies used were rat α-Elav (1:100, Developmental Studies Hybridoma Bank (7E8A10)), mouse α-Repo (1:100, Developmental Studies Hybridoma Bank (8D12)), and chicken α-GFP (1:500, Invitrogen (A10262)). Fluorescently labeled secondary antibodies used were rat Alexa Fluor 633 (1:400, Invitrogen), mouse Alexa Fluor 594 (1:400, Invitrogen), and chicken Alexa Fluor 488 (1:1000, Invitrogen).

### Immunofluorescence quantification

Using ImageJ software, 10-30 repo-positive glial cell nuclei were selected at random from each brain image. >70 glia were selected in total from 5-10 brain images from independent flies for each fly line and condition. All brain images were located in the central brain. GFP expression was quantified using the mean gray value (pixels) of the green channel. The mean gray value was averaged among all selected nuclei of a given age and injury status. Glial cell nuclei were used as a means of quantification because of their relatively low numbers in the *Drosophila* brain, allowing for a more standardized measure of quantification.

### qRT-PCR analysis

Total RNA was extracted from 20 injured or uninjured whole male flies at 4 h post-TBI using Trizol (Invitrogen), according to a modified protocol described by Bogart and Andrews 2006. RNA purification was performed using the RNeasy Mini Kit and RNase-Free DNase (Qiagen). For each sample, 1 µg of RNA was reverse transcribed using the iSCRIPT cDNA synthesis kit (Bio Rad). Quantitative PCR was performed using iTaq Universal SYBR Green SuperMix (Bio Rad) and the Bio Rad CFX96 Real-Time PCR Detection System. Biological replicates of each condition were performed in triplicate and technical replicates were performed in duplicate. Expression of each gene was normalized to expression of *Ribosomal protein L32* (*RpL32*). Primer sequences are shown in Supplementary Table S2.

### Statistical analyses

**Figure 2:**
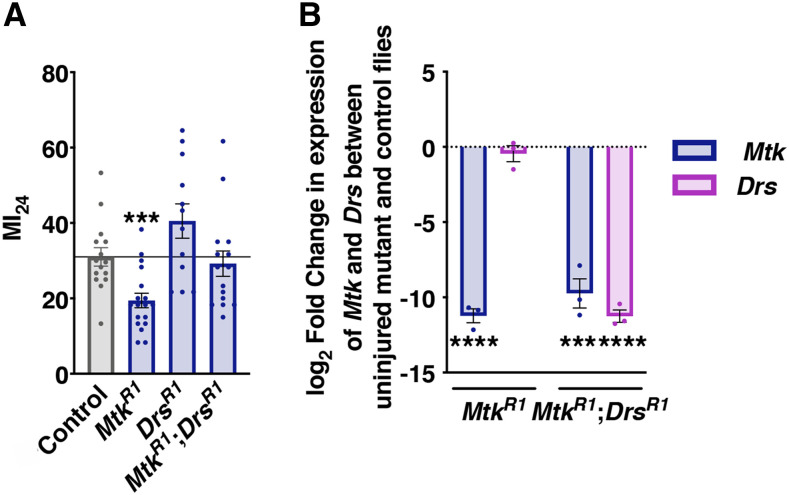
Mutation of *Drs* counteracts the TBI protective effect of mutation in *Mtk*. (A) The MI_24_ of 1-7 day old mixed-sex flies containing mutations in both *Mtk** and **Drs* fed standard molasses food following TBI compared to the MI_24_ of control flies and flies containing a mutation in either *Mtk* or *Drs*. The *Mtk*^*R1*^ and *Drs*^*R1*^ data are from Figure 1A. Each bar represents the average of ≥3 independent trials, and each dot represents the outcome for an individual vial of flies. Error bars represent the SEM. (B) Log_2_ fold change in expression of *Mtk* and *Drs* between 1-7 day old uninjured *Mtk*^*R1*^ and *Mtk*^*R1*^*;Drs*^*R1*^ whole male flies and control flies. All samples were normalized to expression of *RpL32* and statistical testing was performed on ΔCt values (Pfaffl 2001). Each bar represents the average log_2_ fold change in mRNA, and each dot represents the fold change for an individual sample. Error bars represent the SEM (n = 3). Significance was determined by a Student’s unpaired *t*-test. ***P < 0.001, ****P < 0.0001.

**Figure 3:**
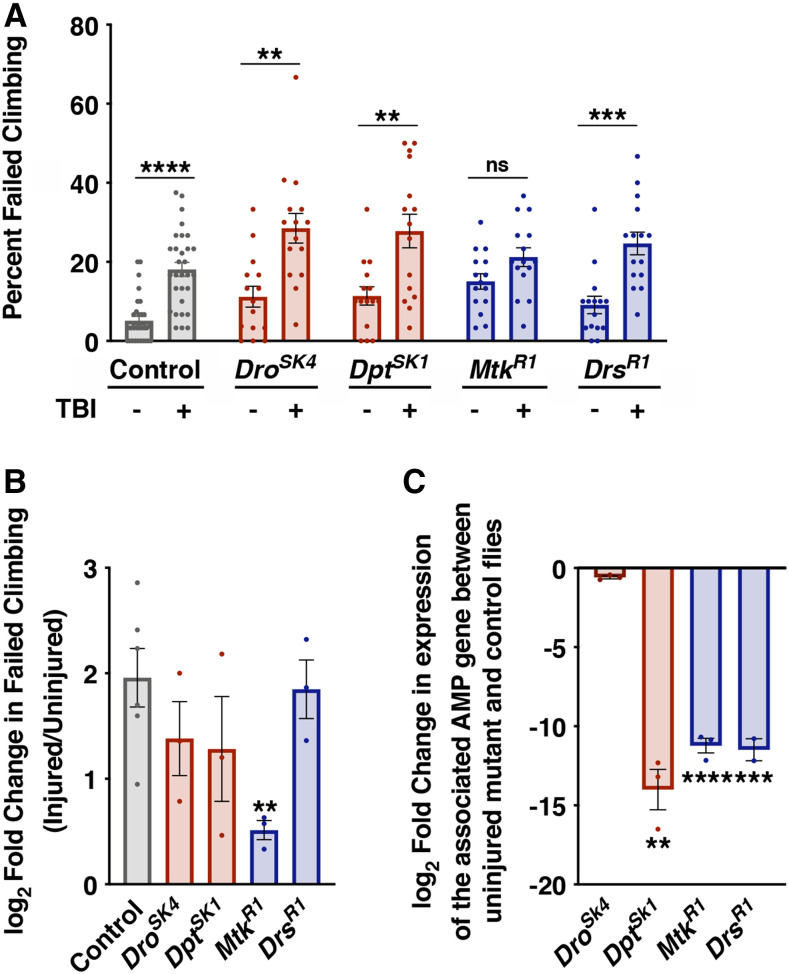
Climbing defects following TBI are reduced in *Mtk*^*R1*^ mutants. (A) Percent of 2-8 day old uninjured and injured flies that failed to complete the climbing task at 24 h following TBI. Each bar represents the average of ≥3 independent trials, and each dot represents the outcome for an individual vial of flies. Error bars represent the SEM. (B) Log_2_ fold change in percent failed climbing between injured and uninjured flies. Each bar represents the average fold change in climbing ability of ≥3 independent trials, and each dot represents the average log_2_ fold change between injured and uninjured flies from a single independent trial consisting of five vials per treatment group. Error bars represent the SEM. Statistical testing was performed on the log_2_ fold changes. (C) qRT-PCR analysis of uninjured whole flies comparing expression of the indicated AMP gene between control and mutant flies. All samples were normalized to expression of *RpL32* and statistical testing was performed on ΔCt values (Pfaffl 2001). The bar and error bars represent the average and SEM, respectively (n = 3). Significance for all panels was determined by a Student’s unpaired *t*-test. *P < 0.05, **P < 0.01, ***P < 0.001, ****P < 0.0001, ns (not significant).

**Figure 4:**
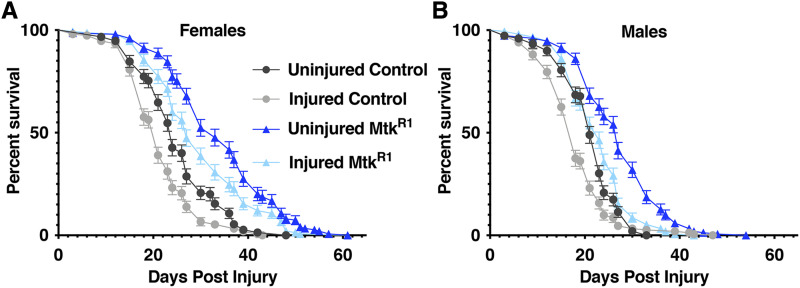
Lifespan of flies with or without TBI is increased in *Mtk*^*R1*^ mutants. Lifespan of 2-8 day old injured and uninjured of (A) female and (B) male control and *Mtk*^*R1*^ flies beginning at 24 h following injury. Error bars represent the SEM of 15 vials of flies from 3 independent trials, n = 150 per condition and sex. Median and maximum lifespan values are provided in Table 1, and statistical analyses are provided in Table S1.

**Figure 5:**
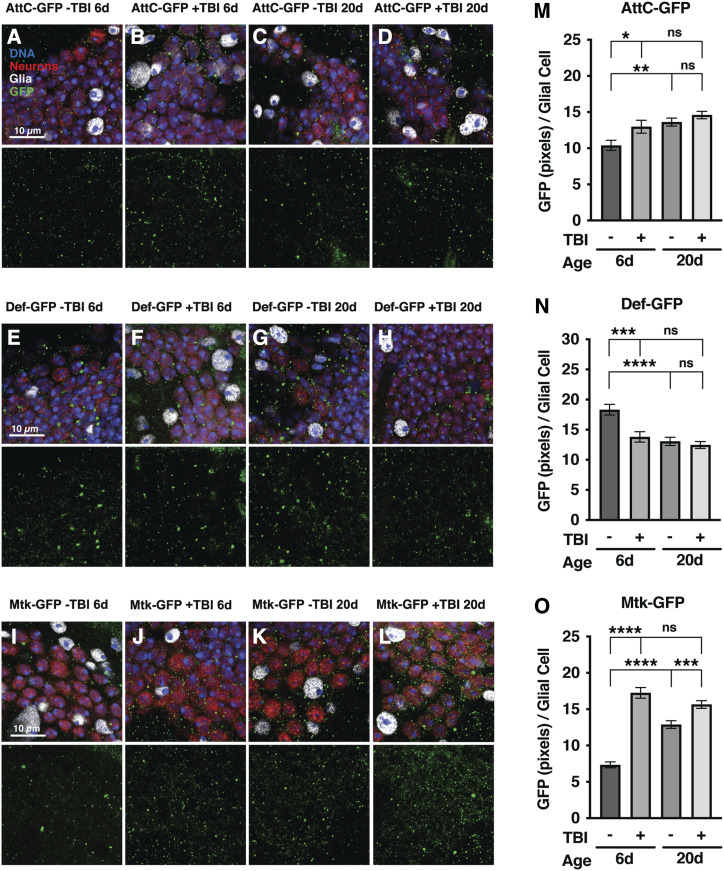
*Mtk* mRNA expression is upregulated in the brain with age and TBI. Representative images of GFP expression (green) in the brain driven by transcriptional regulatory sequences from (A-D) *AttC*, (E-H) *Def*, and (I-L) *Mtk* in younger (6 day old, 6d) and older (20 day old, 20d) flies without (-TBI) and with (+TBI) injury. Brains were stained with the DNA dye DAPI (blue), an antibody to the nuclear neuronal marker Elav (red), and an antibody to the nuclear glial marker Repo (white). Merged images are shown in the top panels, and GFP is shown in the bottom panels. Scale bars represent 10 μm. All samples were collected within 1-4 h post injury, depending on the peak post-injury expression time for each AMP determined in Katzenberger *et al.* 2016 (*AttC*: 3 h, *Def*: 1 h, *Mtk*: 4 h). The amount of GFP expression was quantified as the mean gray value intensity in each glial cell. All bars represent the average GFP expression of (M) *AttC*, (N) *Def*, and (O) *Mtk* in >70 glia measured from 5-10 brains per condition. Error bars represent the SEM. Significance was determined by a two-way ANOVA with Bonferroni post-hoc test. *P < 0.05, **P < 0.001, ***P < 0.001, ****P < 0.0001, ns (not significant).

**Figure 6:**
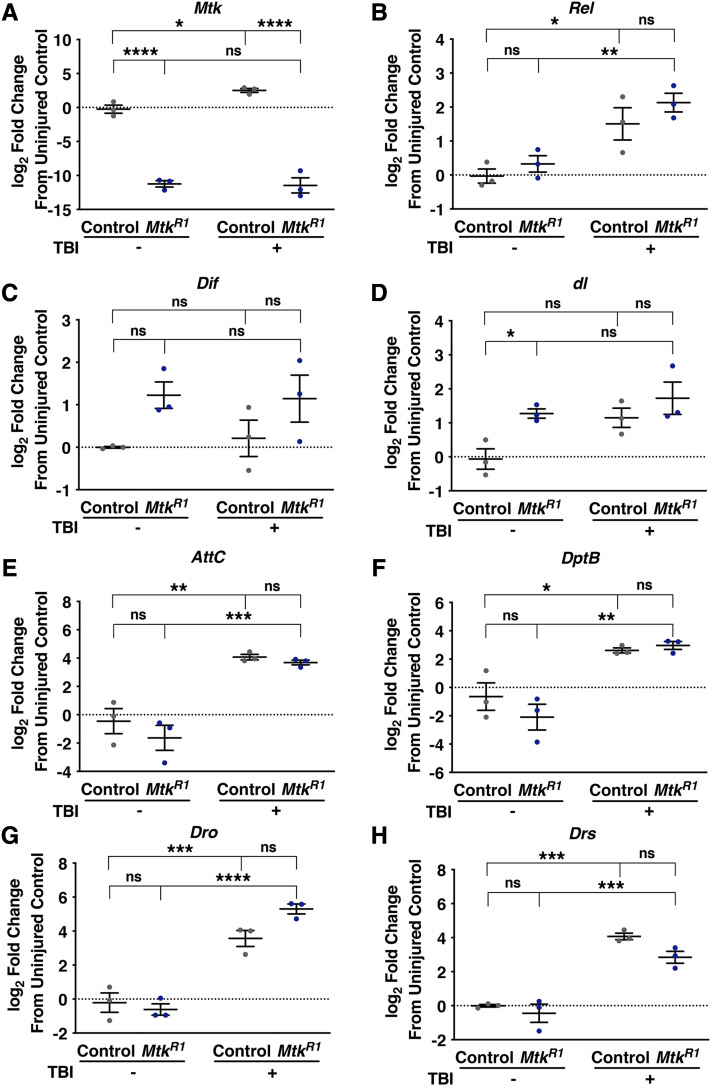
*Mtk*^*R1*^ and control flies similarly activate the Toll and Imd pathways following TBI. qRT-PCR analysis of Imd and Toll pathway genes from uninjured and injured male *Mtk*^*R1*^ and control whole flies collected at 4 h post-TBI. All samples were normalized to expression of *RpL32*. Error bars represent the SEM (n = 3). Significance was determined from ΔCt values by a two-way ANOVA with Bonferroni post-hoc test. *P < 0.05, **P < 0.001, ***P < 0.001, ****P < 0.0001, ns (not significant).

All data are presented as means ± SEM (standard error of the mean). When comparing the outcomes of an AMP mutant line to the control line, a Student’s unpaired *t*-test was used (Figures 1A,1B, 2, and 3). Significance in Figure 1C was determined by a two-way ANOVA with a Bonferroni post-hoc test. Statistical differences in survival were quantified using a log-rank test (Figure 4). For comparisons across multiple groups a two-way ANOVA followed by a Bonferroni post-hoc test was applied (alpha = 0.05, Number of comparisons = 2) (Figures 5 and 6). All statistical analyses were performed using GraphPad Prism 8.

### Data availability

All flies and reagents used in the study will be made publicly available. Supplementary Table S2 contains sequences for all primers used in qRT-PCR analyses. The authors affirm that all other data necessary for confirming the conclusions of the article are present within the article, figures, and tables. Supplemental material available at figshare: https://doi.org/10.25387/g3.12597269.

## Results

### Loss of Mtk reduces acute mortality following TBI

To determine the extent to which AMP genes affect TBI outcomes, we analyzed 10 of the 14 known AMP genes, six single-gene mutations affecting *Attacin C* (*AttC*^*Mi*^), *Attacin D* (*AttD*^*SK1*^), *Defensin* (*Def*^*SK3*^), *Drosocin* (*Dro*^*SK4*^), *Drosomycin* (*Drs*^*R1*^), and *Metchnikowin* (*Mtk*^*R1*^); two small deletions removing both *Diptericins*, *DptA* and *DptB* (*Dpt^SK1^*); and a deletion containing *Dro*, *AttA*, and *AttB* (*Dro**-AttAB^SK2^*) (Hanson *et al.* 2019). Analyses of these lines were compared to a control line (DrosDel iso *w^1118^*) that was used to generate the CRISPR/Cas9 lines and was backcrossed to mutant lines for at least seven generations to generate a uniform genetic background.

Previous studies using our fly TBI model have demonstrated that the percent mortality within 24 h following TBI (MI_24_) is a reproducible measure of the acute TBI response and it correlates with other TBI outcomes such as percent incapacitation and intestinal barrier permeability as well as the median lifespan of uninjured flies (Katzenberger *et al.* 2013; Fischer *et al.* 2018). Using a regular TBI protocol (four strikes from the HIT device spaced 10 min apart), we injured 1-7 day old mixed-sex mutant flies as well as control flies and measured the MI_24_ (Figure 1). Since post-TBI diet alters AMP gene expression and the MI_24_, we assessed the MI_24_ for flies fed a high sugar diet (*i.e.*, cornmeal-molasses food (food)) (Figure 1A) or a low sugar diet (0.4 M sucrose (sucrose)) (Figure 1B) (Katzenberger *et al.* 2013, 2015a 2016). In line with previous studies, all of the lines had a lower MI_24_ with a post-TBI diet of sucrose relative to a diet of food. For flies fed food, only *Mtk*^*R1*^ and *AttD*^*SK1*^ mutants significantly affected the MI_24_ relative to control flies; *Mtk*^*R1*^ reduced the MI_24_ and *AttD*^*SK1*^ increased the MI_24_ (Figure 1A). However, reduced expression of *Mtk* in a line containing null mutations in *Mtk* and *Drs** (Mtk*^*R1*^*;Drs^R1^*) was not sufficient to alter the MI_24_ (Figure 2). Whereas, for flies fed sucrose, *Mtk*^*R1*^, *Dpt^SK1^*, and *AttC*^*Mi*^ mutants reduced the MI_24_, with *Mtk*^*R1*^ having the most significant effect (Figure 1B). Notably, *Mtk*^*R1*^ was the only line for which the MI_24_ was significantly altered under both diet conditions, and in both cases, the MI_24_ was reduced (Figure 1). Thus, expression of the Mtk antimicrobial peptide increases the risk of mortality following TBI. It does so in the context of different diets following TBI (Figure 1), and its effect is counteracted by other AMPs (Figure 2). Since both control and *Mtk*^*R1*^ flies received the same primary injury, the reduced mortality of *Mtk*^*R*^^1^ flies is likely due to reduced secondary injuries.

Because the MI_24_ is affected not only by the diet following TBI but also by the age at which flies are injured (Katzenberger *et al.* 2013), we determined the MI_24_ of older flies. Analyses of mixed-sex 18-24 day old flies showed that the MI_24_ of both control and *Mtk*^*R1*^ flies fed food following TBI significantly increased with age (Figure 1C). However, the MI_24_ of 18-24 day old *Mtk*^*R1*^ flies was not significantly different than that of control flies. Therefore, increased *Mtk* expression, which is known to occur following TBI in both younger and older flies (Katzenberger *et al.* 2016) causes TBI-induced mortality in younger flies but not in older flies, suggesting that targets of Mtk action are not present in older flies, that Mtk-induced events are not necessary to cause mortality in older flies, or that other mortality risk factors are elevated in older flies such that the relative consequence of increased *Mtk* expression is less significant.

### Loss of Mtk reduces behavioral deficits following TBI

To further characterize the role of AMP genes in neurological dysfunction following TBI, we employed a negative geotaxis assay, also called a climbing assay, that is commonly used to measure behavioral deficits in fly models of neurodegeneration (Rhodenizer *et al.* 2008; Ali *et al.* 2011; Petersen *et al.* 2013; Lee *et al.* 2016). We previously found that TBI causes a significant deficit in climbing within 24 h following TBI, with recovery of climbing ability at 48 h post-injury (Katzenberger *et al.* 2013). We analyzed a subset of AMP mutant lines from Figure 1 that represented targets of the Imd (*Dro*^*SK4*^* and Dpt^SK1^*) or Toll (*Mtk*^*R1*^ and *Drs*^*R1*^) pathways and had a range of MI_24_s. We injured 1-7 day old mixed-sex flies using the regular TBI protocol and assayed climbing ability of flies that survived 24 h on food. There was a significant increase in the percent of control flies as well as *Dro*^*SK4*^, *Dpt^SK1^*, and *Drs*^*R1*^ flies that failed to climb following injury compared to uninjured flies (Figure 3A). In contrast, climbing ability of *Mtk*^*R1*^ flies was not significantly different between injured and uninjured flies. To account for differences in baseline climbing ability, we determined the log_2_ fold change in the percent of injured flies that failed to climb relative to uninjured flies that failed to climb for each genotype. The fold change was not significantly different among control, *Dro*^*SK4*^, *Dpt^SK1^*, and *Drs*^*R1*^ flies, but was significantly reduced for *Mtk*^*R1*^ flies (Figure 3B). We used quantitative reverse transcription-PCR (qRT-PCR) to confirm that the mutant AMP genes in these lines were not expressed (Figure 3C). All of these lines had substantially reduced expression of the mutant AMP gene, except for *Dro*^*SK4*^, which contains a single point mutation and a single nucleotide deletion, and should produce a stable mRNA (Hanson *et al.* 2019). These data indicate that elevated expression of Mtk promotes behavioral dysfunction following TBI.

### Loss of Mtk extends the lifespan of injured and uninjured flies

To determine the extent to which loss of *Mtk* alters the long-term effects of TBI, we measured the lifespan of control and *Mtk*^*R1*^ flies injured at 1-7 days old that survived 24 h following TBI. In the absence of injury, both female and male *Mtk*^*R1*^ flies had significantly longer median and maximum lifespans than control flies (Figures 4A and 4B, Tables 1 and S1). The median lifespan of uninjured female and male *Mtk*^*R1*^ flies increased 37% and 24%, respectively. Injured control and *Mtk*^*R1*^ flies had shorter median lifespans than their respective uninjured counterparts, but injured *Mtk*^*R1*^ flies had longer median and maximum lifespans than injured control flies (Figures 4A and 4B, Table 1). For injured flies, the median lifespan of female and male *Mtk*^*R1*^ flies increased 40% and 28%, respectively. Therefore, Mtk negatively affects longevity in both injured and uninjured flies.

**Table 1:** Median and maximum lifespans for Figure 4

	Sex	Fly line	Median lifespan Average ± SEM	Maximum lifespan Average ± SEM
**Uninjured**	Female	Control	24.0 ± 1.0	37.5 ± 1.6
		*Mtk^R1^*	32.9 ± 1.1	50.9 ± 1.2
	Male	Control	21.7 ± 0.4	30.2 ± 0.9
		*Mtk^R1^*	26.8 ± 1.0	38.9 ± 1.5
**Injured**	Female	Control	20.4 ± 0.5	32.3 ± 1.7
		*Mtk^R1^*	28.6 ± 1.2	46.4 ± 1.9
	Male	Control	18.1 ± 0.7	26.7 ± 1.5
		*Mtk^R1^*	23.1 ± 0.8	32.5 ± 1.4

### Mtk expression in the brain is increased by TBI and aging

In flies, both closed-head and penetrating TBI leads to increased expression of AMP genes in fly heads (Katzenberger *et al.* 2016; Ratliff *et al.* 2016; Sanuki *et al.* 2019). However, the source of this expression is not known because fly heads contain multiple cell types, including neurons, glia, and fat body, all of which are known to express AMPs (Cao *et al.* 2013; Kounatidis *et al.* 2017). Therefore, we used immunofluorescence confocal microscopy to examine brain-specific changes in AMP gene expression in the presence or absence of TBI in younger (6 day old) and older (20 day old) flies. We examined expression of a green fluorescent protein (GFP) reporter gene driven by *Mtk* transcriptional regulatory sequences. As a basis for comparison, we also examined reporters for *AttC* and *Def* expression.

AttC-GFP, Def-GFP, and Mtk-GFP expression was detected in neurons and glia in the presence and absence of TBI (Figures 5A-L). Qualitatively, GFP was expressed in both the nucleus and cytoplasm of neurons and glia and uniformly across all regions of the central brain. To quantitate AMP expression, we focused on glia because their large size and low abundance relative to neurons made them easier to analyze, and prior studies implicate AMP gene expression in glia in neurodegeneration (Petersen *et al.* 2012, 2013; Cao *et al.* 2013; Kounatidis *et al.* 2017). AttC-GFP had a small but significant increase in expression in response to TBI in younger flies and aging in uninjured flies (Figure 5M). In contrast, Def-GFP had a significant decrease in expression in response to TBI in younger flies and aging in uninjured flies (Figure 5N). Lastly, Mtk-GFP had a significant increase in expression in response to TBI in both younger and older flies and aging in uninjured flies. However, expression in injured flies did not increase with age (Figure 5O). Qualitatively similar results were observed in neurons (Figures 5I-L). Thus, increased expression of *AttC* and *Mtk* induced by TBI in glia and neurons in the brain may underlie the effects of *AttC*^*Mi*^ and *Mtk*^*R1*^ on mortality following TBI (Figure 1B).

### Mtk mutant flies mount a similar innate immune response as control flies following TBI

Given that Mtk has no known targets outside of its role in combating fungal infection, we considered the possibility that effects of the *Mtk*^*R1*^ mutation following TBI result from effects on expression of other components of the Toll and Imd pathways rather than effects of loss of Mtk molecular activity (De Gregorio 2002; Moghaddam *et al.* 2017). To address the Toll and Imd pathway mechanism, we used qRT-PCR to examine expression of *Relish* (*Rel*), which encodes the NF-κB transcription factor in the Imd pathway; *Dorsal-related immunity factor* (*Dif*) and *dorsal* (*dl*), which encode NF-κB transcription factors in the Toll pathway; and AMP gene targets of the Imd (*AttC*, *DptB*, and *Dro*) and Toll (*Drs*) pathways. We found that TBI caused a significant increase in expression of all of the AMP genes in both control and *Mtk*^*R1*^ flies, with the exception of *Mtk* in *Mtk*^*R1*^ flies (Figures 6F-H). In addition, TBI significantly increased expression of *Rel* in both control and *Mtk*^*R1*^ flies (Figure 6B), whereas expression of *Dif* or *dl* (Figures 6C and 6D) were not affected by TBI. Furthermore, other than *Mtk* expression itself, which was substantially lower in *Mtk*^*R1*^ flies (Figure 6A), in only one case did control flies and *Mtk*^*R1*^ mutant flies show a significant difference in gene expression, and the difference was small (*dl* in uninjured flies (Figure 6D)). Therefore, *Mtk*^*R1*^ flies mount an inflammatory response following TBI that is largely similar to that of control flies, indicating that the beneficial effects on TBI associated with the loss of *Mtk* expression are not due to effects on the Toll and Imd pathways.

## Discussion

### Loss of a single effector of the innate immune response alters the consequences of TBI

In this study, we investigated the influence of individual innate immune response effector genes on development of secondary injuries following TBI. Activation of NF-κB-mediated innate immune response pathways, including Toll-like receptor (TLR) pathways, is one of the earliest and most robust secondary responses to primary injuries in TBI patients and mammalian TBI models (Kumar and Loane 2012; Needham *et al.* 2019). Of the six TLRs in mammals that are expressed on the surface of cells, only TLR2 and TLR4 have been examined in TBI, and mutation of one or both receptors was found to attenuate detrimental outcomes of TBI (Zu and Zha 2012; Laird *et al.* 2014; Jiang *et al.* 2018; Shi *et al.* 2019). Most TBI studies have focused on TLR4, which is expressed on the surface of immune cells and stimulates production of multiple effectors, including the proinflammatory cytokines tumor necrosis factor α (TNFα), interleukin-1β (IL-1β), and IL-6 as well as factors implicated in inflammatory diseases such as inducible nitric oxide synthase (iNOS) and cyclooxygenase-2 (COX-2) (Zhu *et al.* 2014; Jiang *et al.* 2018). Inhibition of TLR4 signaling attenuates neuroinflammation, neural autophagy, and brain edema following TBI, but the extent to which individual TLR4 effectors contribute to these outcomes has not yet been explored. Data presented here suggest that studies of individual effectors will reveal insights that are crucial for understanding how secondary injuries bring about deleterious TBI outcomes.

The *Drosophila* TBI model is well-suited to investigate roles for individual innate immune response effectors because flies have a simpler innate immune system than mammals, but as in mammals, TBI causes rapid and prolonged activation of the innate immune response (Katzenberger *et al.* 2013, 2015a, 2015b 2016; Barekat *et al.* 2016; Sanuki *et al.* 2019). Flies have two major NF-κB-mediated innate immune response pathways: the Toll pathway, which is homologous to mammalian TLR pathways, and the Imd pathway, which is homologous to the mammalian TNFα receptor pathway (Lemaitre and Hoffmann 2007; Bergman *et al.* 2017). Both pathways either directly or indirectly activate transcription of most AMP genes in response to pathogen infection. Moreover, AMPs control biological outcomes that are relevant to TBI in humans. Prolonged expression of individual or multiple AMPs in the absence of pathogen infection results in neurotoxicity (Cao *et al.* 2013; Badinloo *et al.* 2018). Mutations that activate the Imd pathway in the brain lead to neurodegeneration and a shorter lifespan, and overexpression of individual AMP genes in the brain is sufficient to cause neurodegeneration (Tzou *et al.* 2000; Petersen *et al.* 2012; Cao *et al.* 2013; Kounatidis *et al.* 2017; Badinloo *et al.* 2018; Shukla *et al.* 2019). Thus, since TBI in flies activates expression of almost all AMP genes and leads to neurodegeneration and a shorter lifespan, AMPs may be primary effectors of TBI outcomes in flies.

Despite the fact that expression of almost all AMP genes increases shortly after TBI, we found that mutation of only some AMP genes altered the risk of early mortality (Figure 1). When flies were fed cornmeal-molasses food following TBI, *Mtk*^*R1*^ flies had reduced mortality and *AttD*^*SK1*^ flies had increased mortality, while mutations in eight other AMP genes had no effect on mortality (Figure 1A). Thus, activation of the Toll and/or Imd pathway by a primary brain injury triggers expression of both enhancers and suppressors of TBI outcomes. Furthermore, the ability of AMPs to influence TBI outcomes is context-dependent: early mortality was enhanced by *AttD*^*SK1*^ when fed on cornmeal-molasses food but not when fed on 0.4 M sucrose (Figures 1A and 1B); mortality was suppressed by *Dpt^SK1^* and *AttC*^*Mi*^ when fed on 0.4 M sucrose but not cornmeal-molasses food; and mortality was suppressed by *Mtk*^*R1*^ in younger but not older flies (Figure 1C). Since metabolism and aging also affect TBI outcomes in mammals, the potentially complex relationships between individual effectors and metabolism and age in flies likely also occur in mammals and affect the consequences of TBI (Mosenthal *et al.* 2002; Robertson 2004; Shi *et al.* 2016; DeKosky and Asken 2017).

Our data also suggest that additive or synergistic interactions among AMPs determine TBI outcomes. Suppression of mortality by *Mtk*^*R1*^ was abrogated by *Drs*^*R1*^, which as a single mutant had no effect on mortality (Figure 2). Gene expression analyses indicate that the abrogating effect of *Drs*^*R1*^ is not made possible by altered expression of *Drs* or other AMP genes in *Mtk*^*R1*^ flies, suggesting instead that the abrogating effect is due to opposing functions of Mtk and Drs (Figure 6). These apparently counteracting functional interactions among AMPs may underlie our prior observation that reduced expression of multiple AMP genes, including *Mtk*, following TBI by elimination of endogenous bacteria had no effect on mortality (Katzenberger *et al.* 2015a). Thus, it appears that TBI outcomes are determined by the balance of positively and negatively acting effectors of innate immune response signaling pathways.

### An unknown activity of Mtk is detrimental in both TBI and aging

Several lines of evidence suggest that the detrimental effects of TBI and aging involve many of the same physiological processes. In humans, age at the time of TBI is an independent predictor of early mortality and poor short-term recovery, and TBI results in an earlier onset of structural changes to the brain, neurodegeneration, and dementia than in uninjured individuals (Mosenthal *et al.* 2002, 2004; Cole *et al.* 2015; Peters 2016; Liu *et al.* 2017; Fann *et al.* 2018). Aging and TBI are also linked in flies. The median lifespan of uninjured flies of different genotypes negatively correlates with mortality following TBI, TBI-dependent mortality increases with age at the time of TBI, and environmental factors that increase the lifespan of uninjured flies reduce mortality following TBI (Katzenberger *et al.* 2013).

This study supports the idea that the innate immune response mediates at least some of the overlapping pathophysiological outcomes in TBI and normal aging. In flies, the level of activation of the innate immune response, as measured by expression of AMP genes, increases with age (Pletcher *et al.* 2002; Landis *et al.* 2004; Ren *et al.* 2007; Kounatidis *et al.* 2017; Badinloo *et al.* 2018) and the severity of TBI (Figure 6). Moreover, both ubiquitous and fat body-specific overexpression of individual AMPs, including *Mtk*, significantly decrease fly longevity (Badinloo *et al.* 2018). Accordingly, we show here that mutation of *Mtk* not only increased longevity of both injured flies and uninjured flies (Figure 4 and Tables 1 and S1) but also reduced mortality and behavioral deficits following TBI (Figures 1 and 3). Taken together, these data indicate that in wild-type flies the cellular and/or molecular processes controlled by Mtk that reduce longevity also impact behavior and mortality following TBI. Regardless of the nature of this mechanism, its relative importance to TBI outcomes appears to decrease with age, since *Mtk*^*R1*^ reduced mortality in young but not old flies (Figure 1C). The loss of protection in older flies may be due to increased expression of other mortality risk factors, including AMPs, that override the benefits conferred by loss of *Mtk* (Kounatidis *et al.* 2017). The age-dependent effect in *Mtk*^*R1*^ flies is consistent with the findings of Sanuki *et al.* (2019) who demonstrated a protective effect of the antibiotic minocycline following penetrating TBI in young but not old flies. Therefore, processes that mediate aging may also mediate secondary injury-mediated consequences of TBI.

Consistent with our finding that *Mtk* expression increased in the brain with both age and injury (Figure 5), *Mtk* expression in the fly head is associated with neurological dysfunction and neurodegeneration in other contexts (Petersen *et al.* 2012, 2013; Dissel *et al.* 2015; Kounatidis *et al.* 2017; Barajas-Azpeleta *et al.* 2018). Sleep deprivation increases *Mtk* expression in the brain, and overexpression of *Mtk* in neurons or glia is sufficient to disrupt sleep and waking activity (Dissel *et al.* 2015). However, outside of its role as an anti-fungal peptide, little is known about the mechanism by which Mtk signals these changes (Levashina *et al.* 1995; Moghaddam *et al.* 2017). Mtk is a member of the proline-rich family of AMPs that uniquely operate in a selective, nonlytic manner toward pathogens (Scocchi *et al.* 2011). One way that Mtk protects against particular fungi is through inhibition of mitochondrial complex II succinate-coenzyme Q reductase (Moghaddam *et al.* 2017). Assays based on succinate dehydrogenase (SDH) activity showed that Mtk selectively inhibits the SDH activity of different fungi. Possibly, increased SDH activity in the brain of *Mtk* mutant flies reduces the deleterious effects of TBI. This possibility is consistent with the finding that activation of SDH activity in flies prevents neurodegeneration (Van Vranken *et al.* 2014). Alternatively, as has been suggested for other AMPs, Mtk may have a function that is independent of how it responds to pathogens (Cao *et al.* 2013; Petersen *et al.* 2013; Kounatidis *et al.* 2017; Toda *et al.* 2019; Hanson and Lemaitre 2020). A study in *C. elegans* identified a novel role for the anti-fungal AMP NLP-29 as a ligand for the NPR-12 G protein-coupled receptor that signals dendrite degeneration during normal development (E *et al.* 2018). Thus, analogous to NLP-29, Mtk may mediate neural dysfunction and aging through a non-cell-autonomous mechanism. In conclusion, our observations indicate a causative role for Mtk in the propagation of secondary injuries following TBI, possibly as a neuronal signaling molecule.
